# NEP-TC a rRNA Methyltransferase Involved on Somatic Embryogenesis of Tamarillo (*Solanum betaceum* Cav.)

**DOI:** 10.3389/fpls.2019.00438

**Published:** 2019-04-05

**Authors:** Sandra Correia, Ana T. Alhinho, Bruno Casimiro, Célia M. Miguel, Margarida Oliveira, Paula Veríssimo, Jorge Canhoto

**Affiliations:** ^1^Centre for Functional Ecology, Department of Life Sciences, University of Coimbra, Coimbra, Portugal; ^2^Department of Life Sciences, University of Coimbra, Coimbra, Portugal; ^3^Instituto de Tecnologia Química e Biológica António Xavier, Universidade Nova de Lisboa (ITQB-UNL), Oeiras, Portugal; ^4^Centro de Neurociências e Biologia Celular (CNBC/UC), Edifiício da Faculdade de Medicina, Universidade de Coimbra, Coimbra, Portugal

**Keywords:** embryogenic competence, embryogenic callus, gene silencing, rRNA methylation, Solanaceae, SpoU methylase

## Abstract

Somatic embryogenesis (SE) is an important biotechnological tool for large-scale clonal propagation and for embryogenesis research. Moreover, genetic transformation and cryopreservation procedures in many species rely on efficient SE protocols. We have been studying different aspects related to SE induction and somatic embryo development in tamarillo (*Solanum betaceum* Cav.), a small tree from the Solanaceae family. Previous proteomic analyses identified a protein (NEP-TC, 26.5 kDa) consistently present in non-embryogenic calluses of tamarillo, but absent in the embryogenic ones. In this work, the role of NEP-TC during SE was assessed by gene expression analysis and immunolocalization. The results obtained demonstrated that NEP-TC is a putative member of the SpoU rRNA methylase family. This protein, present in the cytoplasm and nucleus, is expressed in non-embryogenic cells and not expressed in embryogenic cells. Slightly enhanced SE induction levels in tamarillo plants with *NEP-TC* down-regulated levels also supports the role of this protein on SE induction. Heterologous expression was used to confirm NEP-TC rRNA methyltransferase activity, with enhanced activity levels when rRNA was used as a substrate. These data relate a putative member of the SpoU methylase family with plant morphogenesis, in particular with SE induction.

## Introduction

Somatic embryogenesis is one of the most useful experimental tools to investigate the morphological, biochemical and physiological events of embryogenesis (Zimmerman, 1993; von Arnold, 2008; Radoeva and Weijers, 2014; De Vries and Weijers, 2017). Several molecular markers associated with the embryogenic competence of cells have been reported and reviewed (Yang and Zhang, 2010; Elhiti et al., 2013; Fehér, 2015), including genes related to cell differentiation, morphogenesis, desiccation tolerance, and signal transduction (Radoeva and Weijers, 2014; Zheng and Perry, 2014; Guan et al., 2016; Horstman et al., 2017; Magnani et al., 2017). Nevertheless, and despite all the efforts, the molecular mechanisms that control the transition of somatic cells with specific functions, into cells capable of forming an embryo, hence expressing totipotency, have been referred as one of the least understood areas of plant developmental biology (Vogel, 2005; Fehér, 2015).

Many embryo-defective mutants have been isolated and analyzed in *Arabidopsis*, allowing a rapid analysis of gene function (Horstman et al., 2017). However, the majority of the genes causing the embryo-defective mutants were related to housekeeping events (e.g., cell division, cell differentiation, phytohormone response and other indispensable survival processes), and only few embryogenesis-specific genes have been found (Ikeda et al., 2006; Radoeva and Weijers, 2014; Horstman et al., 2017). Thus, many aspects of the embryogenesis program remain to be elucidated.

Tamarillo (*Cyphomandra betacea* Cav. syn. *Solanum betaceum*) is a perennial solanaceous in which well-established protocols for somatic embryogenesis induction (Lopes et al., 2000; Canhoto et al., 2005; Correia and Canhoto, 2018) have been developed. The use of biotechnological methods, such as *in vitro* cloning and genetic transformation have also been reported as useful alternatives for tamarillo plant breeding (see Correia and Canhoto, 2012). Furthermore, tamarillo has shown to be a very useful system to better understand specific aspects of *in vitro* morphogenesis, in particular somatic embryogenesis. In this species, zygotic embryos or young leaves are induced on auxin-rich media, originating both embryogenic (EC) and non-embryogenic (NEC) cell lines, which can be separated and grown as independent cell lines in the same culture conditions (Lopes et al., 2000). When transferred to an auxin-free medium, ECs develop into somatic embryos. This makes the species an interesting model for the study of embryogenesis in woody plants since several organs can undergo SE, induced by different auxins, long-term embryogenic tissue can be maintained in culture without losing embryogenic potential, somatic embryo conversion into plantlets is easy and high numbers of plants can be obtained (Correia et al., 2012a). Moreover, SE can be induced in explants derived from adult trees (Correia et al., 2011) and ECs and NECs are obtained from the same tissue. This last aspect is particularly relevant since molecular comparisons can be made between two lines with the same genetic background, but displaying different morphogenic abilities, due to differential gene expression (Correia et al., 2012b).

The comparative analysis of 1D-PAGE of embryogenic and non-embryogenic *calluses* allowed the identification of a 26.5 kDa protein (NEP-TC, Non-Embryogenic Protein from Tamarillo Callus, GenBank accession number JQ766254), which was consistently found in NECs. The presence of this protein in NECs derived from several explants, such as zygotic embryos and isolated hypocotyls, cultured in the same conditions, suggested that it could be considered a good marker for the non-embryogenic cells of tamarillo. In a subsequent work, this protein was isolated, sequenced and identified as a putative RNA methyltransferase (Faro et al., 2003). RNA modification has been extensively studied in bacteria and yeasts, for which most of the biochemical pathways have been identified, in contrast to plants, to which it has rarely been documented (Chen et al., 2010; Ahn et al., 2011).

The main objective of the present work was to characterize the function of the previously identified NEP-TC during somatic embryogenesis induction in tamarillo. To accomplish this objective, *NEP-TC* expression and gene down-regulation assays were performed. The main experimental approaches followed were: (1) *NEP-TC* gene expression analysis and post-transcriptional down-regulation in tamarillo, (2) immunolocalization during the somatic embryogenesis process and (3) heterologous expression to evaluate NEP-TC enzymatic activity. The combination of these methodologies allowed us to discuss the putative role of NEP-TC as a negative regulator of embryogenic potential expression.

## Materials and Methods

### Plant Materials, Growth Conditions, and Tissue Culture

A tamarillo clonal line (TV) was established and propagated from one seedling of a red tamarillo tree, through the successive culture of shoot tips (1.5 cm) in test tubes containing MS basal medium (Murashige and Skoog, 1962) plus 0.07 M sucrose, 0.8 μM benzyladenine (BA), 0.6% (w/v) agar and pH 5.7. Shoot cultures were sub-cultured once a month onto fresh medium of the same composition and kept in a growth chamber at 24 ± 1°C, under 16-h-light cycle with 15–20 μmol m^−2^ s^−1^ illumination. Induction of embryogenic cultures of tamarillo was conducted using the methodology described by Lopes et al. (2000). Leaves excised from *in vitro*-cloned shoots of TV were used as explants. For SE induction the most apical expanding leaves were collected from 6-week-old shoots, randomly punctured and placed abaxial side down in test tubes containing induction medium (IM) - MS basal medium plus 0.25 M sucrose, 20 μM picloram, 0.25% (w/v) phytagel (Sigma) and the pH adjusted to 5.7. Cultures were incubated at 24 ± 1°C under dark conditions during 12 weeks. Induced embryogenic (EC) and non-embryogenic (NEC) calluses were isolated and subcultured at intervals of 4 weeks in the same IM medium. Through all the induction period, samples for subsequent analysis were collected and fast-frozen in liquid nitrogen before storage at −80°C. Somatic embryo-derived plants were obtained by transferring clusters of embryogenic masses to embryo development medium (EDM), MS basal medium plus 0.07 M sucrose, 0.6% (w/v) agar and pH 5.7, under 16-h-light cycle with 15–20 μmol m^−2^ s^−1^ illumination, at 24 ± 1°C, for 8 weeks.

### Phylogenetic Analysis, Multiple Sequence Alignment and Motif Search

Automated gene family annotation resources were used to retrieve the amino acid sequences of NEP-TC homologs from species representative of major plant taxa (Bryophyte, Pteridophyte, Gymnosperms, Angiosperms). Web-based platform Plaza^1^ (Proost et al., 2015) was scanned using NEP-TC predicted amino acid sequence (221 aa). Hand curation with sequence alignment analysis was subsequently used to resolve conflicting results. Phylogenetic analysis was performed using Seaview 4.6.2 (Gouy et al., 2010). First, sequences were aligned with the MUSCLE algorithm v3.8.31 (Edgar, 2004), and maximum likelihood inference was then calculated using PhyML 3.0 (Guindon et al., 2010) with 1000 bootstrap iterations.

The obtained Solanaceae and Brassicaceae protein sequences were selected and multiple sequence alignment was produced using PRofile ALIgNEment (Praline^2^) with default settings (Simossis and Heringa, 2005). Amino acid consistency was calculated by default in Praline, as a linear measure on a scale of 1–10 for consistency in the alignment. To show the similarity between sequences, a conservation output was used where the color scheme represents the conservation level of each residue in the alignment. Conserved PFam motifs were identified with MOTIF^3^. NEP-TC sequence exposed regions were detected by three-dimensional modulation of the protein conformation, using the software Swiss-Model, accessible via the ExPASy web server (Arnold et al., 2006; Larsen et al., 2006). WoLF PSORT prediction^4^ (Horton et al., 2007) was used to predict NEP-TC subcellular localization.

### RNA Extraction and Real-Time Quantitative PCR Analysis

Total RNA was extracted using the RNeasy Plant mini kit (Qiagen, Hilden, Germany) according to the manufacturer’s instructions. RNA samples concentration and quality were determined using a ND-1000 Spectrophotometer (NanoDrop Technologies) and automated electrophoresis with the Experion^TM^ RNA StdSens analysis kit. Reverse transcription was performed according to the 1st Strand cDNA Synthesis Kit for RT-PCR (AMV) form Roche (Mannheim, Germany) using 0.9 μg of total RNA treated with DNaseI (RNase-Free DNase Set, Qiagen, Hilden, Germany). For qPCR, SsoFast EvaGreen Supermix (Bio-Rad) was used in the reaction mixture as per the manufacturer’s instructions, and run in a CFX96 Real-Time System (Bio-Rad). The assay included non-template controls and all reactions were run in triplicate to reduce confounding variance. Data were normalized to the *ELONGATION FACTOR 1-α* (*EF1α*), described as a stable reference gene in potato (*Solanum tuberosum*) during biotic and abiotic stresses (Nicot et al., 2005), with a 380bp fragment amplified by the primer pair: F _EF1α_ 5′-acccgtgaacatgcattgcttgct-3′ and R _EF1α_ 5′-acaccagtctcaacacgaccaaca-3′. PCR primer pair F_NEP_ (5′-acatagcaaagagacacaacgtcggaa-3′) and R_NEP_ (5′-ttgagg- aaggttttagcatcggcaa-3′) was used to amplify a 175bp fragment of *NEP-TC* cDNA sequence. The method used to analyze the qPCR data was the relative quantification method, or 2^−ΔΔCT^ method, where the ΔΔCT value = [(CT_1_Target − CT_1_Reference) – (CT_0_Target − CT_0_Reference)] (Livak and Schmittgen, 2001). The mean CT values for both the target and internal reference genes were determined and the fold change in the target gene normalized to *EF1α* and relative to the expression in the control sample.

### Protein Extraction, Separation and Immunoblot Analysis

Total protein extraction was performed as previously described (Correia et al., 2012b), from 0.35 to 0.5 g of leaves, induced explants and EC samples and from 1 g of NEC samples, that were grounded to a fine powder in liquid nitrogen. While still frozen, the powder was suspended in 4 ml of cold acetone containing 0.2% (w/v) dithiothreitol (DTT) and 10% (w/v) trichloroacetic acid (TCA). The suspension was then transferred to clean centrifuge tubes, incubated overnight at −20°C, and centrifuged for 30 min at 20,000g (4°C). The supernatant was carefully decanted, and the remaining protein pellet was washed twice in cold acetone (containing 0.2% DTT), incubated for 30 min at −20°C, centrifuged 30 min at 20,000 *g* (4°C), and vacuum-dried. The resulting pellet was resuspended in 500 μL of solubilization buffer [7 M urea, 2 M thiourea, 2% (w/v) CHAPS (3[(3-cholamidopropyl)dimethylammonio]-propane- sulfonic acid), 1% (w/v) DTT]. The suspension was sonicated and incubated at room temperature for 2 h in a rotary shaker and then centrifuged at 20,000 *g* for 1 h to remove the insoluble material. Total protein concentration was assessed using the 2-D Quant Kit (GE Healthcare; Amersham Biosciences) according to the manufacturer’s guidelines and using BSA as standard. Samples were aliquoted and stored at −20°C until further processing. Proteins were resolved by 10% SDS-PAGE. To each aliquot of 20 μg of the solubilized proteins, 2% SDS and bromophenol blue were added before separation. Prestained Precision Protein Standards (Bio-Rad) were used as molecular weight markers (250–10 kDa). Proteins resolved by SDS-PAGE (gels not stained) were electroblotted onto polyvinylidene fluoride (PVDF) membranes (Millipore), in transferring buffer (CAPS, *N*-cyclohexyl-3-aminopropanesulfonic acid 10% methanol), overnight, at 4°C, 40 V. The membranes were incubated in TBS-T (Tris-HCl Buffered Saline-Tween) buffer with 5% (w/v) skim milk (blocking solution) for 45 min at room temperature, and then with the rabbit anti-NEP-TC policlonal antibody diluted to 1:1500 in blocking solution, for 1 h at room temperature (Jacek et al., 1992). This policlonal antibody was raised against the peptide antigen IPQYGCGTASLN, corresponding to residues 123–134 of an exposed region of NEP-TC (Genosphere Biotechnologies). Membranes were then placed in the SNAP i.d. Protein Detection System (Millipore), washed four times with TBS-T, with vacuum running continuously, and incubated with an alkaline phosphatase-conjugated anti-rabbit IgG monoclonal antibody (Sigma-Aldrich), diluted to 1:5000 with a blocking solution, for 10 min at room temperature. Detection was performed by enhanced chemifluorescence technique (GE Healthcare). NEC protein extracts were used to verify serum specificity by comparison with pre-immune serum reaction, and antibody titer.

### Immunohistochemistry

The localization of NEP-TC expression on embryogenic and non-embryogenic tissues was based on the method described by Casson et al. (2005), modified for highly hydrated material. Briefly, the material was fixed in a solution of ethanol and acetic acid (3:1, v/v) during 48 h at 4°C, with agitation, followed by dehydration by immersion in different solutions with increasing concentrations of sucrose (10, 15, and 34%) on PBS buffer pH 7.4. Each treatment was applied for 48 h at 4°C with agitation. After dehydration, the material was frozen in molds using O.C.T (Tissue-Tek^^®^^) and immersed in liquid nitrogen. Sectioning was carried out using a Leica CM3050S cryostat. The histological sections (14 μm thick) were fixed on microscope slides covered with poly-L-lysine (Thermo Scientific).

Immunohistochemical analysis was based on the method described by Sauer et al. (2006). Tissue permeation was ensued by incubation at 37°C for 30 min, with a 2% (w/v) driselase solution, followed by washing with PBS pH 7.4 solution during 5 min, and an incubation with blocking solution (10% BSA in PBS pH 7.4) for 1 h, at room temperature. Sections were subsequently incubated with a 1:10 solution of primary antibody overnight on a wet chamber. The antibody dilution was performed in PBS buffer, pH 7.4, with 1% BSA (w/v). After the first incubation, the sections were washed again with PBS pH 7.4, and incubated with the secondary antibody Alexa Fluor 633 (Thermo Fisher) during 1 h in the wet chamber. After incubation, the material was covered with DAKO fluorescent mounting medium and cover slips. Control samples were prepared by omitting the antibodies in the incubation solutions. The observations were performed in a confocal Zeiss LSM510 META model microscope, with 621 nm excitation and 639 nm emission.

Image analysis was performed using ZEN software (Zeiss), Version 2.1, as well as Fiji Image J (Schneider et al., 2012). The values of integrated density determined for the control without antibody labeling were subtracted to the ones obtained for the labeled tissues.

### Ultrastructure and Immunogold Labeling of NEP-TC

For studying EC and NEC cells ultrastructure the samples were fixed for 2 h, at room temperature (RT), in a 3% (v/v) formaldehyde, 0.5% (v/v) glutaraldehyde, 2% (w/v) sucrose and 0.05% (w/v) CaCl_2_ solution prepared with 2.5% NaPIPES buffer, pH 7.2, followed by three 10 min washes with the same buffer. Samples were thoroughly dehydrated in an ethanol series: 10%, 10 min at RT; 20%, overnight at RT; 30%, 30 min at 4°C; 50%, 30 min at 4°C; 70%, 20 min at −20°C; 90%, 30 min at −20; 100%, 30 min at −20°C. Samples were embedded in LR White resin (Sigma), at −20°C. LR White capsules with the embedded tissues were polymerized at 55°C overnight. Ultrathin sections (70 nm) were obtained with a Leica EM UC6 ultramicrotome (Leica Microsystems) and collected on formvar-coated nickel grids. The grids were placed in 0.05 M glycine for 20 min followed by phosphate-buffered saline (PBS) with 5% bovine serum albumin (BSA) for 30 min at room temperature and then incubated for 2 h with the primary antibody diluted (1:100) in PBS containing 5% BSA. The sections were washed three times in PBS and incubated with the secondary antibody (goat anti-rat coupled with 20-nm colloidal gold, BioCell International) diluted 1:200 in PBS supplemented with 1% BSA. The grids were washed in PBS and distilled water and dried at 37°C. Ultra-thin sections were stained with uranyl acetate followed by lead citrate. Observations were carried out on a FEI-Tecnai G2 Spirit Bio Twin at 100 kV.

### Vector Construction and Plant Transformation

GATEWAY^TM^ cloning technology (Invitrogen) was used to directionally insert a specific fragment of *NEP-TC* into a destination vector, pK7GWIG2(I) obtained from UGent – VIB Research (Belgium). A 264 pb PCR fragment of the *NEP-TC* cDNA was made GATEWAY-compatible with template-specific primers (with attB1 and attB2 sequences) and amplified by PCR using the designed gene-specific forward and reverse primers (F 5′-tgccgatgctaaaaccttcc-3′; R 5′-ccagcccaaactccaaat-3′). The T-DNA region of this vector contains a negative selectable marker (ccdB gene) to select against non-recombinant clones, promoter and terminator of 35S, the GATEWAY cassette (two inverted AttR regions separated by an intron) and the neomycin phosphotransferase II gene (nptII) as the plant selectable marker. PCR and *in vitro* BP and LR Clonase (Invitrogen) recombination reactions were carried out according to the manufacturer instructions (Supplementary Material S1). The obtained recombinant plasmid was electroporated into LBA4404 *Agrobacterium tumefaciens* competent cells. *A. tumefaciens* was grown on Luria broth (LB) medium with appropriate antibiotics (rifampicin 50 μg/ml and spectinomycin 50 μg/ml).

One-month-induced leaf explants of TV tamarillo plantlets were used as explants for transformation. Transformation medium (TfM) was composed of liquid IM and the bacterial suspension supplemented with 20 μM acetosyringone, in a proportion of 1:50. The explants were wounded with a scalpel, immersed in TfM for 5 min, rinsed thoroughly in sterile water and blotted dry on sterile filter paper before a 2-day cocultivation period, in dark conditions, on induction medium (IM) regeneration plates with 20 μM acetosyringone. After cocultivation, explants were rinsed in a MS salt solution with carbenicillin (250 μg/ml) and cefotaxime (250 μg/ml) and blotted dry on sterile filter paper, before being transferred to IM plates with carbenicillin (250 mg/l), cefotaxime (250 μg/ml) and kanamycin (100 mg/l). Explants were maintained in the dark, at 24°C, and transferred to fresh IM selective plates with 14 days. After 8–12 weeks, kanamycin-resistant embryogenic cells were transferred to selective EDM, with 50 μg/ml kanamycin, and plants obtained from somatic embryos as described above. All the experiments were followed by controls treated as described above except for the presence of antibiotics in the culture media.

The transgenic nature of the plants was confirmed by amplification of a 700 bp fragment of the *nptII* gene (F 5′-gaggctattcggctatgactg-3′ and R 5′-atcgggagcggcgataccgta-3′) (Supplementary Material S1). Genomic DNA was extracted using the DNeasy^^®^^ Plant Mini kit (Qiagen). *NEP-TC* down-regulation was verified by qPCR as described above. Somatic embryogenesis competence and shoot and root development were evaluated in transformed plants.

### Recombinant NEP-TC Methyltransferase Activity Analysis

#### Vector Construction and Protein Expression

NEP-TC cDNA sequence was subcloned into the expression vector PET-28b expression plasmid which was transformed into *Escherichia coli* BL21 competent cells. The successful cloning of NEP-TC cDNA was verified by sequencing. Transformed cell colonies were first grown in 5 ml of LB medium with kanamycin (30 mg/ml), at 37°C, and then 4 mL of cell suspension were added to the 500 ml of LB medium, and cell growth proceeded for a period of 5 h, at 37°C with agitation. Target protein expression was induced by 1 mM IPTG at 30°C overnight.

#### Cell Lysis and Recombinant NEP-TC Purification

After induction, cells were collected by centrifugation (5,000 *g*, 4°C). The pellet re-suspended in 4 ml of lysis buffer (2 M urea, 0.05 M Tris and 0.3 M NaCl, pH 8), to which 45 ml of a second lysis buffer (0.1% Triton X-100, 1 mm DTT, supplemented with 100 μl of DNAseI and 100 μL of MgCl_2_). Purification of the protein from the lysate was carried out by ion-metal affinity chromatography (IMAC). The lysate was incubated with the matrix for 1 h at 4°C. After incubation, the matrix was subjected to successive elutions with increasing concentrations of Imidazole (20, 50, 200, and 500 mM) for removal of the purified protein. Afterwards, a molecular exclusion chromatography was performed using a Superdex 200-PG column 160 cm × 15 cm (GE Healthcare Life Sciences), utilizing a renaturation buffer (0.25 M Tris-HCl, 0.15 M NaCl, pH 8.5). The protein fractions collected during the molecular exclusion chromatography were concentrated using a centricon filter unit (Supplementary Material S2). The final protein concentration was determined with the Bradford method (Bradford, 1976). The purified protein was tested by denaturing SDS-PAGE and western blot analysis (methods described above).

#### Substrates Isolation

Total DNA extraction from non-embryogenic calli was performed using the NucleoSpin Plant II kit (Macherey-Nagel), while the extraction of total RNA from the same tissues was performed using the kit NucleoSpin RNA Plant (Macherey-Nagel). The quantification of this substrates was performed in a NanoDrop spectrophotometer. Total RNA quality was verified by gel electrophoresis and automated electrophoresis analysis with Experion^TM^ RNA StdSens Analysis kit (Bio-Rad). An agarose gel was prepared (1% agarose, 1x TBE buffer in DEPC treated water, Midori Green staining solution). The rRNA fraction was extracted from the agarose gel using the High Pure PCR Product Purification kit (Roche). Ribosome purification was performed according to the method described by Rivera et al. (2015) with slight modifications. NEC samples (13 g FW) were grinded to a fine powder in liquid nitrogen and resuspended in 2 volumes of plant extraction buffer (50 mM Tris-HCl pH 9, 30 mM MgCl_2_, 400 mM KCl, 17% sucrose). The homogenate was then passed through two layers of cheesecloth and the crude lysate layered over a sucrose cushion buffer (20 mM Tris-HCl pH 7.6, 5 mM MgCl_2_, 50 mM NH_4_Cl, 60% sucrose) and centrifuged at 10°C for 3 h. The resulting pellet was suspended in 160 μL of re-suspension buffer (50 mM KCl, 20 mM Tris-HCl pH 7.6, 5 mM MgCl_2_) and stored at −80°C for further analysis. Ribosome concentration was calculated according to the literature (Meskauskas et al., 2005), by establishing the correspondence between 1 unit of absorbance at 260 nm (A260) and 20 pmol of plant ribosomes, corresponding to 0.54 pmol/ml.

#### Enzyme Activity Assays

Enzymatic characterization of recombinant NEP-CT was performed using the CBA096 SAM Methyltransferase Assay (Calbiochem-Merck Millipore) kit, which is based on the measurement of the absorbance resulting from the production of SAH in SAM-dependent methylation reactions. The final product of this reaction (H_2_O_2_) is measured by interaction with the colorimetric agent. The absorbance was measured at 510 nm in a Spectra Max Plus 384 spectrophotometer. All the assays were performed in duplicate in micro well plates. In the assays performed recombinant NEP-TC had a concentration of 714 μg/ml, while the substrates, total DNA and total RNA, had concentrations corresponding to 222.14 μg/ml and 238.80 μg/ml, respectively. In further assays, the substrates, corresponding to rRNA and ribosomes, had a final concentration of 46.00 and 0.54 μmol/ml, respectively. Nucleic acid concentration was estimated by using the conversion factors of 1 A260 dsDNA = 50 μg/ml and 1 A260 ssRNA = 40 μg/ml.

### Statistical Analysis

Statistical analysis (GraphPad version) was performed by Students *t*-test or analysis of variance (ANOVA) and significantly different means were identified using the Tukey test (*p* < 0.05).

## Results

### NEP-TC Is a Putative Member of the SpoU rRNA Methylase Family

**FIGURE 1:**
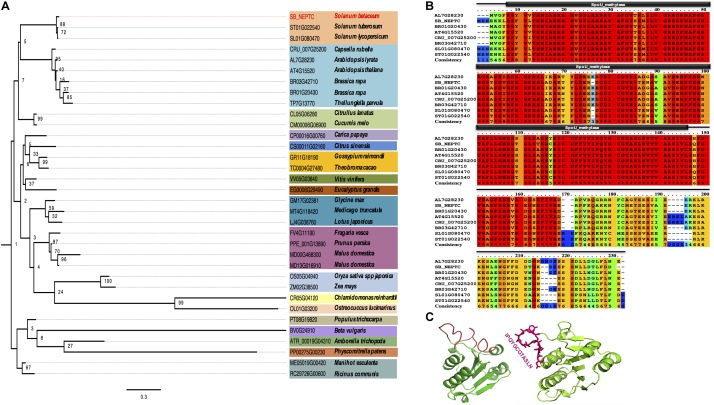
Tamarillo NEP-TC sequence alignment and similarity relationships of the most closely related proteins **(A)** Neighbor-joining tree of *S. betaceum* NEP-TC (SB_NEPTC) HOM03D005634 family of proteins in plant and algae species. Proteins of the NEP-TC family from a selection of 34 species representing all major Viridiplantae taxa were used in the phylogenetic analysis. The phylogenetic tree was constructed using maximum-likehood and bootstrap values from 1000 replicates. Numbers on each branch represent the percentages of bootstrap. Conserved domain analysis of *S. betaceum* NEP-TC (SB_NEPTC). **(B)** Multiple sequence alignment of conserved regions matching significant protein motifs (SpoU_methylase, PF00588) and domains of NEP-TC and homologs from the Brassicaceae [*Arabidopsis lyrata* (AL7G2830), *Brassica rapa* (BR01G20430 and BR03G42710), *Arabidopsis thaliana* (AT4G15520), *Capsella rubella* (CRU_007G2500)] and Solanaceae [*Solanum lycopersicum* (SL01G080470), *Solanum tuberosum* (ST01G022540)] families. Amino acid consistency was classified from 0 (unconserved) to 10 (conserved); perfect amino acid match in all species is indicated with an asterisk. Gaps (–) were introduced to maximize alignment. **(C)**Three-dimensional modulation of NEP-TC conformation using the software Swiss-Model to predict the structure and the exposed domains (red loops), including the peptide IPQYGCGTASLN against which a polyclonal antibody was made.

To determine the phylogeny of NEP-TC, a protein expressed in non-embryogenic tissues of tamarillo, homologs were retrieved from web-based comparative genomics platforms (Plaza Dicots 3.0) and hand curated, generating a set of 34 known sequences from proteins expressed in other plant and algae species. Those full protein sequences were aligned and subsequently used for phylogenetic inference (Figure 1A). The outgroup consisted of one sequence per major taxon (Chlorophyta, Bryophyta, Monocots, and Eudicots) of the HOM03D005634 family. No members of this gene family were observed in the genomes of Pteridophytes or Gymnosperm species. From a common root we observed the branching out of two clades, with the most diverse clade including NEP-TC form *Solanum betaceum* and several Eudicots species, as well as representatives from the Monocots (*Oryza sativa* and *Zea mays*) and green algae (*Ostreococcus lucimarinus* and *Chlamydomonas reinhardtii*). The minor clade included the Bryophyta *Physcomitrella patens* and Eudicot species.

Multiple sequence alignment of *S. betaceum* NEP-TC and the homologs from other Solanaceae (*Solanum lycopersicum* and *S. betaceum*) and Brassicaceae species showed a high percentage of conserved domains between residues 10 and 150 (Figure 1B), in line with the MOTIF identification of a conserved domain of the SpoU rRNA Methylase family (PF00588, *E*-value 4.1e^−28^), a family of proteins with RNA methyltransferase activity and RNA binding and processing functions which may use *S*-AdoMet (*S*-adenosyl-L-methionine) as methyl donor. In *Arabidopsis thaliana*, this protein family includes at least five different proteins that share the conserved domain SpoU_MeTrfase (IPR001537), found in RNA methyltransferases TrmH, and also in NEP-TC. Bioinformatics analysis of *NEP-TC* cDNA (666 bp) and amino acid (221 aa) sequences gave important insights about the putative role of this protein. ProtParam analysis showed that NEP-TC encodes 221 amino acids with a theoretical mass of 24.74 kDa and a theoretical isoelectric point of 6.09. 3D modulation of NEP-TC (Figure 1C) was a useful tool to better visualize the exposed regions of the protein and to select the most appropriate and specific sequences for polyclonal antibody production. The selected peptide was a predicted exposed region, which includes a set of amino acid residues displaying high antigenicity that enhances antibody-antigen reaction. NEP-TC has a globular structure, characterized by the presence of an alpha/beta knot, as well as a tRNA (guanine-N1-)-methyltransferase N-terminal, characteristics that also predict that this protein is a tRNA/rRNA methyltransferase of the SpoU rRNA Methylase family. With WoLF PSort software we could predict the NEP-TC subcellular location as most likely to be cytoplasmatic (0.85 likelihood) with no nuclear localization signals.

### NEP-TC Protein and Gene Expression During SE Induction in Tamarillo

**FIGURE 2:**
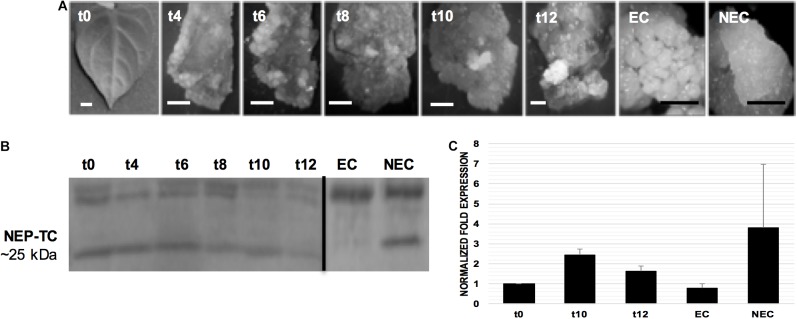
Somatic embryogenesis (SE) induction in tamarillo leaf explants of tamarillo TV line and NEP-TC protein and gene expression analysis. **(A)** Leaves (week t0) and induced leaves (weeks t4–t12) were removed from the induction medium at 2-week intervals after the 4th week of culture for protein and gene expression analysis. embryogenic (EC) and non-embryogenic (NEC) calluses were obtained after the 12 weeks induction period, and sub-cultured separately for 1 month before analysis. Bars: 1 mm. **(B)** Western blotting of proteins extracted from induced, EC and NEC samples. Twenty micrograms of extracted protein were loaded into each well for SDS-PAGE, blotted, and reacted with the antibody raised against NEP-TC exposed domain. **(C)** Expression of *NEP-TC* in the late stages of SE induction, and calluses stages, measured using qPCR. Relative expression was calculated using the ΔΔCT method and normalization was made to *EF1α* expression using t0 time point as the control. Bars represent standard deviation (*n* = 3).

The SE induction on tamarillo leaf explants was conducted using the methodology described by Lopes et al. (2000). On the induction medium, the young leaves underwent a dedifferentiation process (Figure 2A), after which two types of calluses, embryogenic (EC) and non-embryogenic (NEC), were formed. Calluses started to appear by the 6th week of culture and, 2–3 weeks later, areas of whitish, compact embryogenic cell masses could be distinguished among the more friable and fast growing non-embryogenic calluses. These small embryogenic areas were isolated from the surrounding non-embryogenic calluses and subcultured in the same culture medium, without losing their multiplication and embryogenic potential.

To determine NEP-TC expression during the SE induction process, a specific polyclonal antibody was produced. Immunoblots from leaf-derived (Figure 2B) explants, indicated that the expression of a protein of ∼25 kDa is only absent on EC. In fact, besides NEC samples, the 25 kDa protein is also expressed in the initial explants and earlier induction stages and its expression decreases when embryogenic areas start to appear on the induced explants. The data also showed that the antibody identified proteins with other molecular weights, in particular a ∼32 to 35 kDa protein, present both in EC and NEC, but more abundant in ECs where the ∼25 kDa band was absent.

The qRT-PCR analysis (Figure 2C) confirmed the expected high levels of *NEP-TC* transcripts in NEC samples (around 3.3 twofold differential gene expression compared to t0). EC samples showed the lowest gene expression levels, and the latest time points of SE induction (t10 and t12), in which explant dedifferentiation is the highest, showed increased levels (2.4 and 1.6 twofold change, respectively) compared to differentiated leaves in t0.

### NEP-TC Is a Cytosolic and Nuclear Protein Expressed in NEC Cells

**FIGURE 3:**
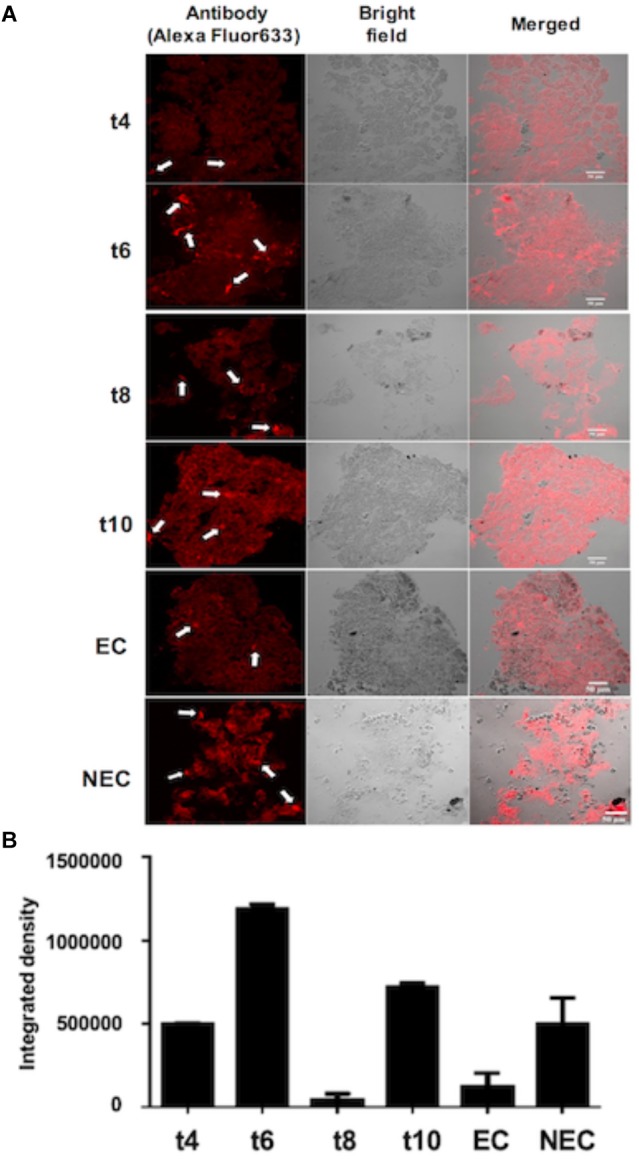
Immunolocalization of NEP-TC in histological sections (14 μm) of tamarillo leaf explants cultured for 4 weeks (t4) to 10 weeks (t10) of culture, and embryogenic (EC) and non-embryogenic (NEC) calluses. **(A)** Sections stained with the anti-NEP-TC antibody and Alexa Fluor 633 (left column). Sections observed under transmission light (center column) were used for histological control. Arrows indicate the places where the protein has been more intensivelly labeled. **(B)** Integrated fluorescence results for the immunolocalization assays.

In order to determine NEP-TC localization in the embryogenic and non-embryogenic tissues of tamarillo, as well as in the dedifferentiated tissues, sections were obtained and incubated with anti NEP-TC antibody (Figure 3A). Analyzing the images corresponding to the proembryogenic tissues, it is clear that 4 weeks after the induction, NEP-TC appears in some distinct labeled spots, that increase in frequency along the induction phase (Figure 3A), reaching its peak in the 6th week. The obvious exception to this increasing tendency is the 8th week, where the labeling is reduced. However, it increases consistently after, in the 10th week of development (Figure 3B). When it comes to the non-embryogenic callus significant labeling was observed in the whole tissues’ extension, whilst in embryogenic tissue the presence of NEP-TC was not frequent, or even absent, thus showing that the immunolocalization results are consistent with those from the Western blot. A closer look at the non-embryogenic cells that presented labeling with the antibody allowed to observe NEP-TC in the peripheral area of the cells (Figure 3).

**FIGURE 4:**
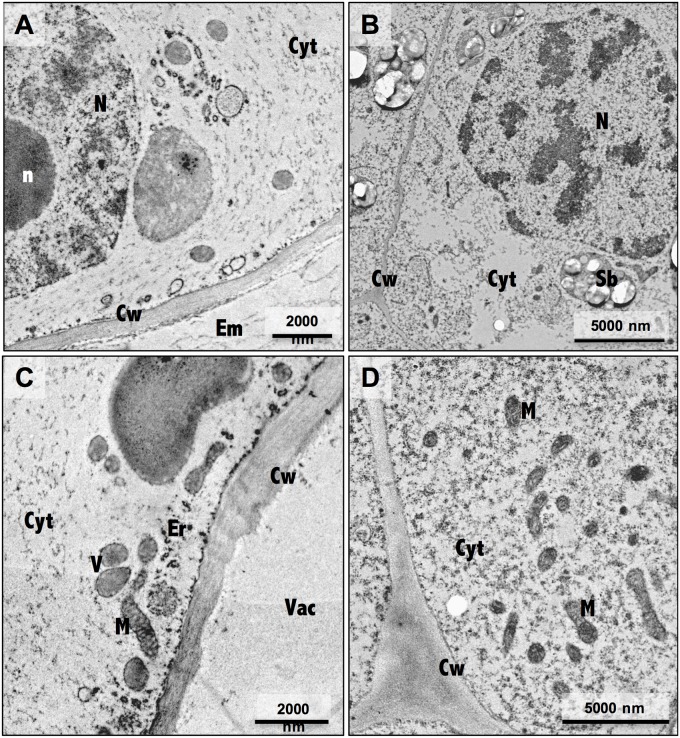
Electron micrographs of non-embryogenic **(A,C)** and embryogenic **(B,D)** cells. N, nucleus; n, nucleolus; Cw, cell wall; Em, extracellular matrix; Cyt, cytoplasm; Vac, vacuole; M, mitochondria; Sb, starch bodies.

**FIGURE 5:**
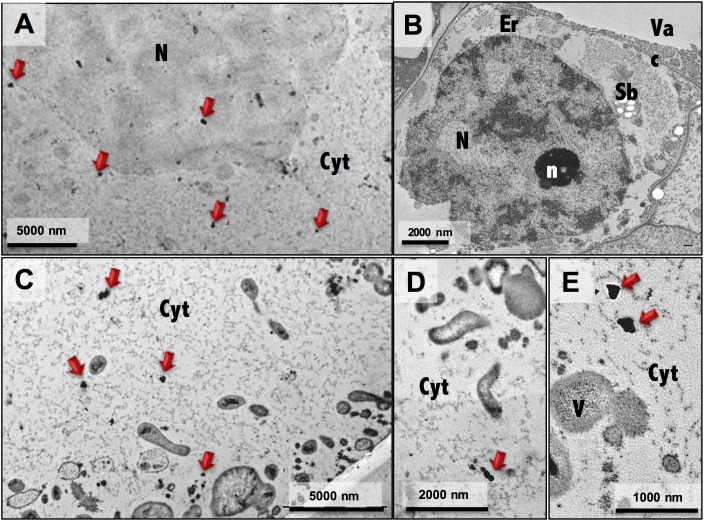
Immunogold labeling of NEP-TC epitope on EC and NEC cells. General views of a non-embryogenic cell **(A)** and embryogenic cells **(B)** with NEP-TC labeling in the cytoplasm and nucleus (red arrows) of NEC cells. More detailed electron micrographs **(C–E)** of NEC cells showing clusters of immunolabeled NEP-TC more abundantly detected in the cytoplasm. N, nucleus; n, nucleolus; Cw, cell wall; Em, extracellular matrix; Cyt, cytoplasm; Vac, vacuole; M, mitochondria; Sb, starch bodies.

Ultrastructure analysis showed pronounced differences between embryogenic and non-embryogenic cells (Figure 4), namely the undifferentiated characteristics of NEC cells, such as a large central vacuole and thin peripheral cytoplasm with minimal number of organelles (Figure 4A,C), and the meristematic-like features of EC cells, with small vacuoles and a densely cytoplasm with a large variety of organelles (Figure 4B,D). Combining this ultrastructural analysis with immunogold labeling of NEP-TC epitopes on both samples it was possible to detect NEP-TC presence mostly in the cytoplasm but also in the nucleus of NEC cells (Figure 5A), and never in EC cells (Figure 5B). In NEC cells the protein appeared in small clusters in the center of the cells or near small vesicles in the cell periphery (Figure 5A,C–E) and sometimes similar clusters appeared in the nucleus (Figure 5A).

### Tamarillo Lines With *NEP-TC* Down-Regulation Show Slightly Enhanced SE Induction

**FIGURE 6:**
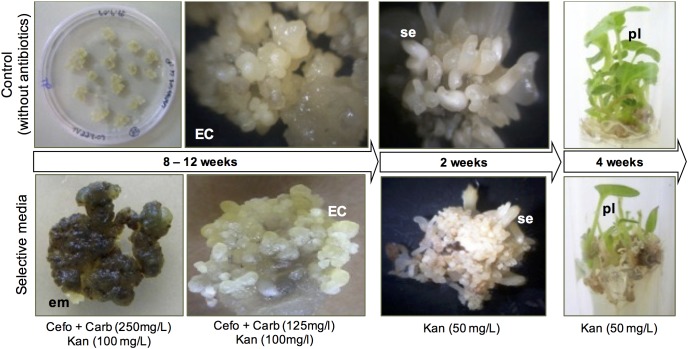
Post-transcriptional gene silencing of NEP-TC gene in tamarillo with regeneration of kanamycin-resistant plants through somatic embryogenesis. All the regeneration process of transformed explants was followed by a control in which no antibiotics were added to the culture medium, to insure the embryogenic ability of the explants used. EC, embryogenic callus; em, embryogenic masses; se, somatic embryos; pl, plants.

To evaluate the role of NEP-TC in tamarillo SE, a post-transcriptional gene-silencing strategy was used to down-regulate *NEP-TC* expression in tamarillo plants. For this purpose, a 264 bp PCR fragment of *NEP-TC* cDNA sequence, was inserted via recombination into pK7GWIWG2(I), a vector that generate hpRNA constructs. PCR and restriction analysis with HindIII confirmed the construction before incorporation into *A. tumefaciens* LBA4404 strain. Tamarillo lines with *NEP-TC* down-regulation were then obtained by an *A. tumefaciens*-mediated genetic transformation protocol and regeneration through SE (Figure 6). Agrobacterium-mediated transformation using 1-month induced explants was found to be suitable to produce large numbers of independently transformed transgenic lines in tamarillo. Simultaneous application of a selection pressure (presence of a set of 3 antibiotics) combined with embryogenic induction conditions were found to be suitable for the production of kanamycin-resistant clumps of embryogenic cells, within 10–12 weeks (Figure 6). Nevertheless, induction rates were very low, only 2 from a total of 106 explants (an average of 21 explants in 5 replicate treatments), co-cultivated with Agrobacterium, developed embryogenic tissues. The obtained kanamycin-resistant embryogenic masses were carefully isolated from the surrounding non-resistant tissues and placed in fresh induction selective medium, where they proliferated, hence increasing their mass (Figure 6). These kanamycin-resistant embryogenic masses developed into somatic embryos after 2–3 weeks in EDM selective medium. Also, the putative transgenic embryos were grown into plantlets (Figure 6), using a protocol similar to that followed for regeneration of normal plantlets but containing kanamycin (50 μg/ml) in the medium. A total of 80 kanamycin-resistant somatic embryo-derived plantlets developed on selection medium, in test tubes, from an initial 20 mg mass of resistant embryogenic callus, and 75% of these were positive for *nptII* gene expression.

**FIGURE 7:**
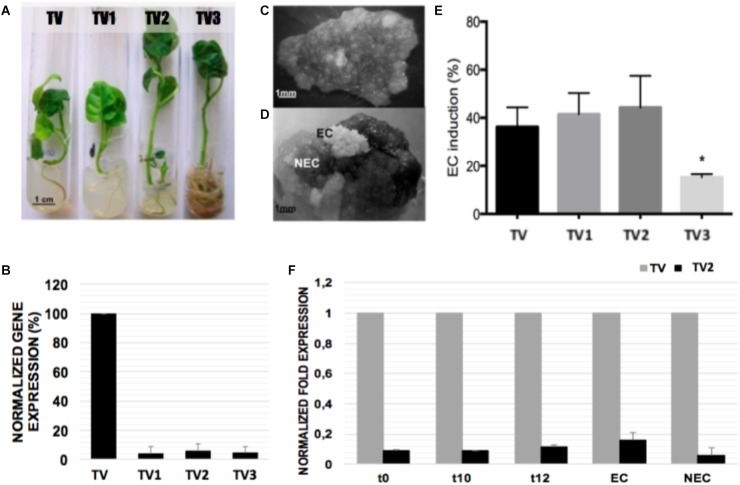
Somatic embryogenesis induction and gene expression analysis in leaf explants of tamarillo NEP-TC down-regulated lines. **(A)** 3 transformed lines (TV1, TV2, and TV3) and in a control plant (TV) were randomly selected. **(B)** qRT-PCR analysis of the NEP-TC transcript levels showed efficient gene silencing (around 85%). The expression of *EF1α* was used to normalize gene expression and TV CNE gene expression values used as control. Expression values are the result of 3 replicates ±SD. **(C,D)** Somatic embryogenesis was induced in leaf explants of the lines tested. The explants reacted in similar ways for control and down-regulated plants, and after 12 weeks in culture, embryogenic (EC) and non-embryogenic (NEC) areas were evident. **(E)** Percentage of EC formed per total of explants induced showed slightly higher values for TV1 and TV2 lines even though with no significant differences using the Tukey test (*p* < 0.05). **(F)** qRT-PCR (same conditions as previously described) time-course analysis of NEP-TC expression for line TV2 (higher SE levels).

qRT-PCR (Figure 7A) was used to compare *NEP-TC* expression levels in three randomly selected transformed lines (TV1, TV2, and TV3), together with a control plant (TV). The abundance of transcripts encoding NEP-TC was substantially reduced in the down-regulated lines that showed approximately 85% reduction in transcript expression (Figure 7B,F). Nevertheless, no consistent phenotypic differences were observed between the down-regulated and the control plants, except for the more accentuated development of adventitious roots in line TV3 (Figure 7A). Even in terms of somatic embryogenesis induction, leaf explants from the transformed lines responded in a similar way when compared to the control line (Figure 7C,D), and even though no significant differences were observed between the percentage of ECs formed in the control line and the percentage formed by the down-regulation lines, lines TV1 and TV2 showed slightly increased levels of EC induction (Figure 7E).

### Recombinant NEP-TC Methyltransferase Activity Is Enhanced With rRNA as a Substrate

**FIGURE 8:**
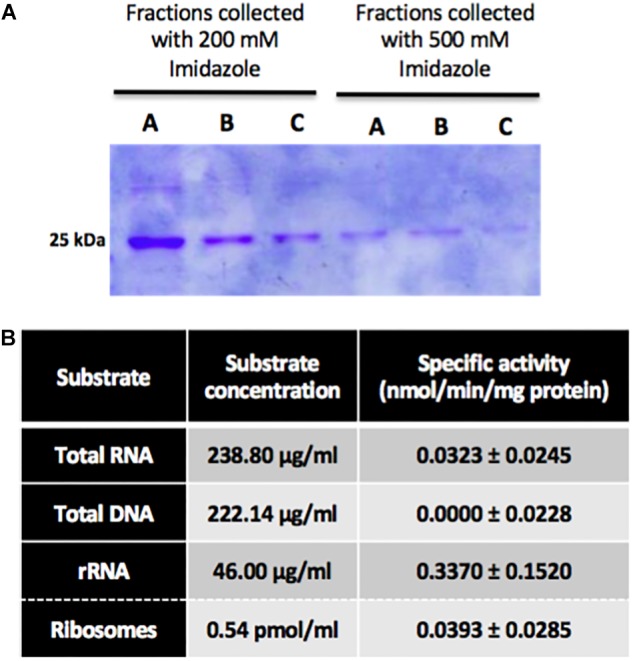
Somatic embryogenesis induction and gene expression analysis in leaf explants of tamarillo NEP-TC down-regulated lines. **(A)** SDS-page results of successive protein fractions (A, B, and C) collected with 200 and 500 mM of imidazole after the molecular exclusion chromatography of the dialized samples, showing NEP-TC band around 25 kDa. 20 μL of each sample were loaded in the wells. **(B)** Chart with NEP-TC’s specific activity in the presence of different substrates. The results showed were obtained in three biological replicates, except for DNA samples that showed no specific activity.

In order to perform enzymatic assays to determine NEP-TC’s specific activity, the recombinant protein was expressed on a heterologous system. Thus, *E. coli* cells were transformed with a plasmid encoding the NEP-TC gene, the protein’s expression was induced with IPTG and, after cell lysis, several chromatographies and a dialysis were performed in order to isolate it. 500 ml of expression culture yielded approximately 2.048 mg of protein, which corresponds to 4.1 mg of protein per liter of expression culture. The protein fractions collected with 200 and 500 mM of imidazole showed higher purity levels, and therefore selected to be used in enzymatic activity analysis, being pooled and concentrated. The protein fractions collected after the molecular exclusion chromatography were analyzed by SDS-PAGE (Figure 8A). The dialysis was necessary in order to evaluate the protein’s stability and to guarantee the buffer exchange for the kinetic activity assays. Quantification of the recombinant protein in the dialyzed pool fractions by Bradford assays shows that it has an average concentration of approximately 714 μg/ml. This purified protein was used in Calbiochem’s SAM Methyltransferase Assay kit to determine its affinity toward different substrates. The concentrations of total DNA and total RNA used in the assays corresponded to 222.14 and 238.80 μg/ml, respectively. Since the bioinformatic data shows that this protein might be a rRNA methyltransferase, enzymatic assays were performed with both ribosomes isolated from non-embryogenic calli and rRNA extracted from total RNA isolated from the same tissue. The concentration of rRNA corresponds to 46.00 μg/ml, whereas the ribosome concentration corresponds to 0.54 pmol/ml. The specific activity values for these substrates are summarized in the chart in Figure 8B. According to the results displayed, it is possible to observe that NEP-TC has higher specific activity values toward total RNA, with an average value of 0.0323 nmol/min/mg protein. There was no specific activity toward DNA, which allowed the elimination of DNA as a potential substrate, and the subsequent assays were performed in order to determine which fraction of the total RNA this protein methylates. It was possible to determine that the NEP-TC does in fact methylate rRNA with specific activity values (0.3370 ± 0.152 nmol/min/mg protein) ten times higher when compared to total RNA specific activity values (0.0323 ± 0.0245 nmol/min/mg protein).

## Discussion

The present work was carried out to evaluate the putative role of a protein previously identified in non-embryogenic *calluses* of tamarillo that has been named NEP-TC (GenBank accession number JQ766254). The full length cDNA of *NEP-TC* was previously isolated and sequenced from a non-embryogenic callus library (Faro et al., 2003) as a result of a comparative analysis of SDS-PAGE profiles of ECs and NECs of tamarillo.

The predicted 221 amino acid protein encoded by *NEP-TC* contains a significant sequence similarity to proteins of the SpoU Methylase family, which is the second largest group of RNA methyltransferases (MTases). All of these enzymes are dimers, with the catalytic site formed at the interface of two monomers, which modify RNA by a methyl transfer reaction in which *S*-adenosyl-methionine (SAM) is the donor of the methyl group. Although methylation of nucleic acids by SAM occurs spontaneously, MTases enhance the reaction rates by promoting favorable orientation and spatial proximity between RNA and the methyl donor group (Motorin and Helm, 2011). RNA methylation is important for biophysical stabilization of the RNA structure (Lapeyre, 2005) and previous reports suggest that rRNA and tRNA anticodon loop methylations might have a role in the fidelity of mRNA decoding by the ribosome (Decatur and Fournier, 2002). More recent reports referred that hypomodified tRNA species may result from the cellular response to oxidative stress conditions, as described for tRNA methylation in *Saccharomyces cerevisiae* (Chan et al., 2010). These observations reinforce the idea that the dynamic nature of RNA post-transcriptional modifications may be crucial for the control of gene expression. The results obtained in this work associate a putative member of the SpoU Methylase family protein with the somatic embryogenesis induction process, which is a morphogenic process strongly associated with stress conditions (Zavattieri et al., 2010; Takáč et al., 2011), as also suggested in tamarillo through the identification of several stress-related proteins (Correia et al., 2012b).

By analyzing NEP-TC expression in more detail throughout the induction process in leaves, it was found that, contrarily to what was previously observed (Faro et al., 2003) NEP-TC is not exclusively expressed in NECs. Immunoblot analysis shows that the protein is expressed in the early stages of SE induction (until week 8) starting to diminish afterwards, and is completely absent in ECs. Despite these observations, *NEP-TC* transcript analysis revealed expression levels also in ECs.

To evaluate the role of this putative RNA methyltransferase in tamarillo somatic embryogenesis, a functional genomics approach was adopted, trying to assess gene function through its down-regulation or silencing. In what concerns the targeted gene silencing in this work, a qPCR analysis of the randomly selected transformed plants, showed high levels of *NEP-TC* down-regulation (around 85% silencing) in several lines. Despite the obtained down-regulation levels, only slightly enhanced somatic embryogenesis induction rates could be observed when compared to the control plants. The absence of evident phenotypes related to *NEP-TC* down-regulation seems to indicate that other proteins in this family may have redundant functions in somatic embryogenesis. Immunoblot analysis strongly suggests a temporal expression pattern of different members of a putative SpoU MTases family.

The difficulty in relating individual genes to phenotypes (Wang and Waterhouse, 2001), and the substantial gene redundancy that many plant gene families present (Busov et al., 2010), have already been referred as relevant limitations for the dissection of individual gene function(s) through functional genomics approaches as the ones used in this work. Genetic redundancy is a major problem for dissecting gene function in all plant species. Therefore, single loss-of-function mutations often do not have obvious phenotypic effects in most experimental environments, which could explain the results in tamarillo.

The results obtained show that NEP-TC could not only be a member of an important regulatory protein family of RNA MTases, but also a relevant key regulator for somatic embryogenesis in tamarillo. Recently, more attention is being devoted to modifications in newly discovered RNA species, and some authors sustain that these changes could establish the links to various cellular mechanisms including gene silencing, transcriptional modulation of gene expression, stress response and, possibly, development and epigenetics (Motorin and Helm, 2011).

The results obtained confirmed the predicted cytosolic location of NEP-TC, by the prevailing observation of NEP-TC labeling in the cytoplasm of NEC cells, but also showed that it can be located in the cell nucleus. Data available in the literature concerning the subcellular localization of RNA methyltransferases in plants is scarce or even absent. Even though pre-rRNA processing including 2′-*O*-methylation has been known to occur in nucleolus, several rRNA modifications occur on the later stages of rRNA maturation, particularly on the smaller subunit of eukaryotic ribosomes, just before ribosome assembly. Those later small modifications are very important for translation stability and the cellular localization is still unclear in many cases (reviewed by Sloan et al., 2017).

Even though several recombinant rRNA methyltransferases have been expressed in heterologous systems, the data concerning the protein yield of the whole process are usually not mentioned in the papers’ results. Agarwalla et al. (2002) reported a successful expression of a recombinant rRNA methyltransferase in *E. coli* with a final concentration of 2.5 mg of purified protein per liter of expression culture. Comparing these reference values with the ones obtained for NEP-TC, it is clear that the whole optimized process used for the expression of this protein is more advantageous, as it permitted the obtainment of a considerably higher concentration of purified protein. Considering this process’ high yield, the present methodology used for NEP-TC’s expression can continue to be used for further characterization of this enzyme. Concerning NEP-TC’s specificity all reactions performed with RNA substrates showed methylation activity, with rRNA showing consistently higher values. Thus, based on these results we can confirm NEP-TC activity as a rRNA methyltransferase, even though NEP-TC dual-specificity as a rRNA/tRNA methyltransferase cannot be ruled out, as reported for other rRNA methyltransferases (Ahn et al., 2011; Sharma et al., 2014). A possible RNA substrate flexibility associated with NEP-TC would be important to elucidate for a complete understanding of the biological function of these enzyme, and although several rRNA methyltransferases have been identified in plants, there is virtually no information about the physiological role of these enzymes. However, in bacteria and yeast, they are frequently expressed as a response to abiotic stress, like nutrient restriction or exposure to antibiotics (Liu et al., 2008; Sharma et al., 2014). The induction of SE is a process which conditions comprise several stress factors to the plant cell. Since the induction of SE in tamarillo is a process in which the explants are exposed to osmotic stress, wounding and contact with picloram (an herbicide), it can be hypothesized that NEP-TC influences the response of cells to stress conditions and further acquisition of embryogenic competence through mechanisms of post-transcriptional regulation.

Several genes have been reported as being involved in the process of somatic embryogenesis induction, like *LEC* (*LEAFY COTYLEDON*) (Gaj et al., 2005) or *SERK* (*SOMATIC EMBRYOGENESIS RECEPTOR- LIKE KINASE*) (Aker et al., 2006), both being considered essential for the acquisition of embryogenic competence. As far as we know, no inhibitory genes related to the induction of somatic embryogenesis have been reported before. The molecular mechanisms through which the expression of the NEP-TC gene can negatively interfere with somatic embryogenesis are not known. A decreased methylation activity would make tRNA and rRNA less stable, thus compromising the efficiency of the translational process. It is known that protein synthesis is critical for somatic embryogenesis induction (Takáč et al., 2011), a situation that could also be observed in tamarillo (Correia et al., 2012b). Also, the examination of differentially expressed proteins between tamarillo ECs and NECs suggested that the embryogenic cells could have a better ability to regulate the effects of stress conditions, namely, through the action of heat-shock proteins (HSPs), in contrast with NEC cells in which HSPs expression was not detected (Correia et al., 2012b). Thus, the expression of RNA MTases, such as NEP-TC points to a necessity of protein synthesis stabilization in NEC cells. In this context, the hypothesis that NEP-TC could be a RNA MTase associated with non-embryogenic stages of the somatic embryogenesis process seems reasonable.

## Conclusion

The involvement of a protein family of RNA MTases in the somatic embryogenesis induction process was evaluated. NEP-TC is a putative member of the SpoU rRNA methylase family in tamarillo, with a potential role in somatic embryogenesis regulation, namely in embryogenic competence acquisition. The few data available about rRNA modifications, particularly methylation, in eukaryotes emphasizes the role of this mechanism in rRNA stabilization during ribosome assembly, allowing an efficient translation process. It is known that protein synthesis control is critical for somatic embryogenesis induction, and active protein synthesis could also be observed in tamarillo. Understanding the molecular regulation of somatic embryogenesis induction may help to develop the necessary tools to optimize these protocols and also to improve regeneration/transformation systems in woody species. In this way, the search for molecular markers of critical stages of somatic embryogenesis is a promising field of research, and the optimization of somatic embryogenesis protocols for an efficient use in woody species is of utmost importance. Further studies are necessary to verify the involvement of RNA MTases in other embryogenic systems as well as to clarify what are they targets and how they act to prevent the expression of the embryogenic potential.

## Author Contributions

SC participated in the design of the study, acquisition and analysis of data for the work, performed the statistical analysis, and drafted the manuscript. AA and BC participated in acquisition of the data. CM and MO participated in the design and coordination of the study and revising it critically for important intellectual content. PV participated in the design and coordination of the study and revising it critically for important intellectual content. JC conceived the study, participated in its design and coordination, revising it critically for important intellectual content, and final approval of the version to be published. All authors read and approved the final manuscript.

## Conflict of Interest Statement

The authors declare that the research was conducted in the absence of any commercial or financial relationships that could be construed as a potential conflict of interest.

## Footnotes

1 http://bioinformatics.psb.ugent.be/plaza/

2 http://www.ibi.vu.nl/programs/PRALINEwww/

3 https://www.genome.jp/tools/motif/

4 https://wolfpsort.hgc.jp
