# Supplementary material for: NEP-TC a rRNA Methyltransferase Involved on Somatic Embryogenesis of Tamarillo (Solanum betaceum Cav.)
Source: Front Plant Sci. 2019 Apr 5;10:438. doi: 10.3389/fpls.2019.00438 (PMC6459958; doi:10.3389/fpls.2019.00438)
Supplement: Supplementary file 1 [file Data_Sheet_1.PDF]

Supplementary Material S1

Agrobacterium-mediated genetic transformation of tamarillo explants with plant regeneration through somatic embryogenesis

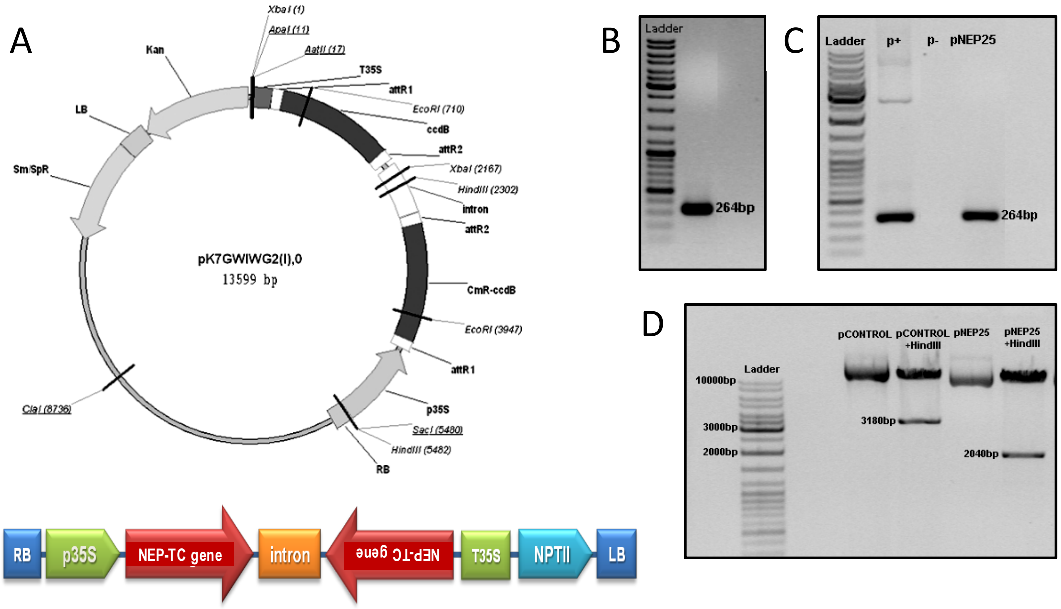

Fig. 1. Post-transcriptional gene silencing of *NEP-TC* gene in tamarillo. **(A)** Construction of a GATEWAY vector (Invitrogen). The final product of recombination contains one spliceable intron and *nptII* as plant selectable marker gene, and produces double-stranded RNA - hairpin RNA - from the *NEP25* gene sequence, triggering post-transcriptional gene silencing. **(B)** NEP-TC PCR products were generated with attB1 and attB2 recombinant sites, producing a 264bp fragment. **(C)** **(D)** The construct sequence was verified by PCR and restriction analysis, before being incorporated into the *Agrobacterium* strain.

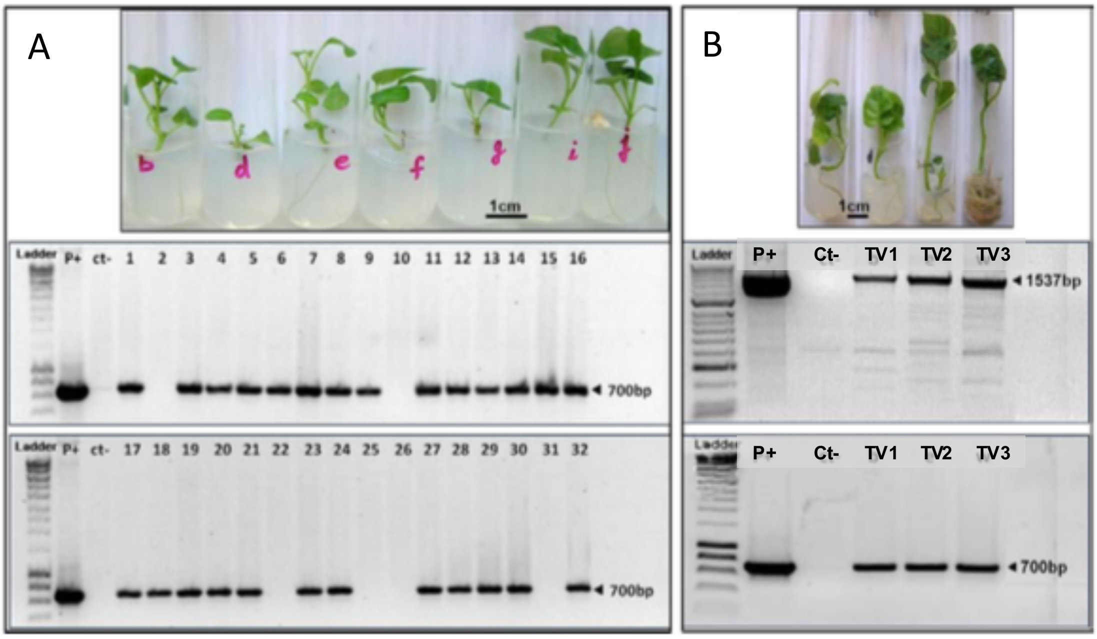

Fig. 2. Electrophoretic analysis of the PCR products of 36 putative transgenic tamarillo plants. **(A)** PCR-screening of almost half of the 80 self-rooted plants obtained, through the amplification of a 700bp fragment with primers for nptII. Some of the plants inside the test tubes are showed. **(B)** Analyses of 3 selected putative transgenic plants (lines TV1, TV2 and TV3). The upper gel shows the amplification of a 1537 bp fragment, with primers for the construct intron and P35S. In the lower gel, a 700bp fragment from nptII is amplified. The positive control was p*NEP-TC* plasmid (p+) and non-transformed plants were used as negative control (ct-).
