# Supplementary material for: NEP-TC a rRNA Methyltransferase Involved on Somatic Embryogenesis of Tamarillo (Solanum betaceum Cav.)
Source: Front Plant Sci. 2019 Apr 5;10:438. doi: 10.3389/fpls.2019.00438 (PMC6459958; doi:10.3389/fpls.2019.00438)
Supplement: Supplementary file 2 [file Data_Sheet_2.PDF]

Supplementary Material S2

Recombinant NEP-TC expression and purification

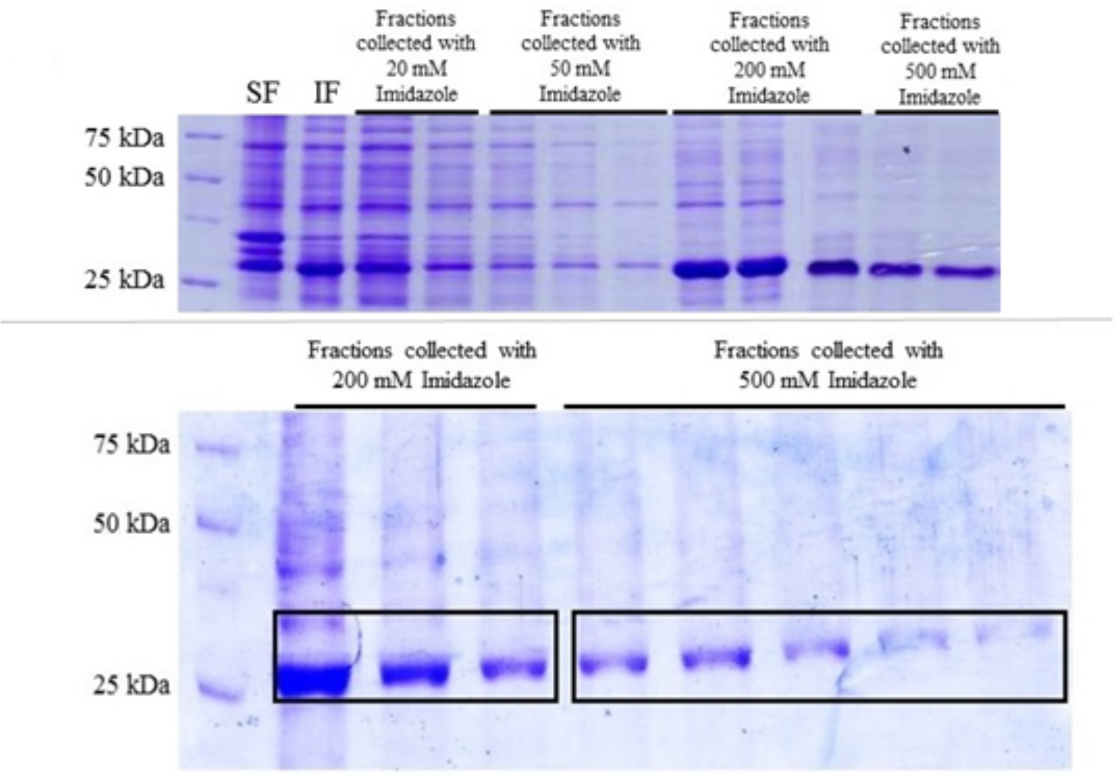

Fig. 1. NEP-TC fractions collected from the IMAC column. (23A) “SF” and “IF” correspond to the proteins’ soluble and insoluble fractions. (23B) Detail of the fractions collected with 200 and 500 mM Imidazole. The boxes comprise the strips that correspond to approximately 25 kDa. 20µL of each sample were loaded in the wells. it is possible to see the fractions collected from the ion-metal affinity chromatography., the purified protein matches the 25 kDa strips, and NEP-TC molecular weight is 26.5 kDa. It is also possible to see some protein aggregation in the first purified fractions, in the 50 kDa and 75 kDa strips. It is also clear that the protein has a higher concentration in its soluble form.

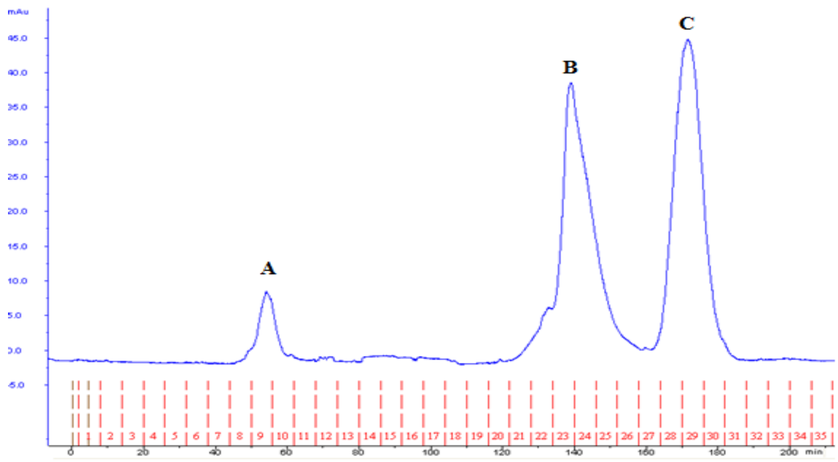

Fig. 2. Gel filtration results. The first peak (A) corresponds to NEP-TC in its aggregated form, while the second peak (B) corresponds to the protein on its purified form. The third peak (C) corresponds to Imidazole.
